# Inadvertent Traumatic Fracture of Central Venous Catheter during Procurement, Transmitted Through Solid Organ Transplant

**DOI:** 10.1155/2018/5406098

**Published:** 2018-05-30

**Authors:** Thaer Obaid, Corinne Cricco, Ani Simka, Richard Fine, Saravanan Ramamoorthy, Radi Zaki

**Affiliations:** ^1^Department of Transplant Surgery, Einstein Healthcare Network, Philadelphia, PA, USA; ^2^Department of Anesthesiology, Einstein Healthcare Network, Philadelphia, PA, USA

## Abstract

Central venous catheters play a pivotal role in the perioperative support of critically ill patients. They are used for administration of fluids, vasopressors, blood products, and various medications; however, their use may be associated with serious complications, such as catheter fracture and embolization. While most data on catheter fracture embolization consist of isolated case reports, only a few studies have examined patients with central venous catheter embolism. We report a traumatic inadvertent transection of central venous catheter that migrated through a donor transplanted liver and was found to be lodged in the recipient's right ventricle. The catheter was retrieved under fluoroscopy using a trilobed snare device.

## 1. Introduction

Percutaneous central venous access is a common practice in hospitals and outpatient facilities. It is estimated that more than 5 million central lines are inserted per year in the United States for various purposes, including hemodynamic monitoring, renal replacement therapy, nutritional support, and medication administration [1]. Complications of central venous catheter insertion are reported to be about 11% of total number of catheters placed and include structural disruption associated with technical difficulties with catheter placements, infection, and thrombotic events. Catheter fracture and embolization is a rare, but serious complication, occurring at an estimated rate of 0.1–0.3% [2]. Most of the reported fractures occurred in implantable central venous ports due to compression of the catheter between the clavicle and the first rib, also known as “Pinch-off syndrome” [3].

We present a unique case report of an inadvertent traumatic transection of central line catheter during organ procurement that migrated to the recipient with subsequent embolization to the right ventricle following liver transplant reperfusion.

## 2. Case Report

A 66-year-old female with a past medical history of nonalcoholic steatohepatitis, complicated by ascites, hepatorenal syndrome, and spontaneous bacterial peritonitis, with a MELD (Model for End-Stage Liver Disease) score of 31, was admitted for an orthotopic liver transplant.

General anesthesia was induced with fentanyl, propofol, and succinylcholine. An 8.0 Endotracheal tube was placed in the trachea. A vecuronium drip at 4 mg/hr was started. Invasive monitoring consisted of a 20 gauge, 10 cmin length arterial catheter placed in left radial artery. A right internal jugular 7-French 16 cm in length triple lumen central venous catheter and a 16-French SVC catheter were inserted under ultrasonographic guidance using Seldinger technique.

**Figure 1:**
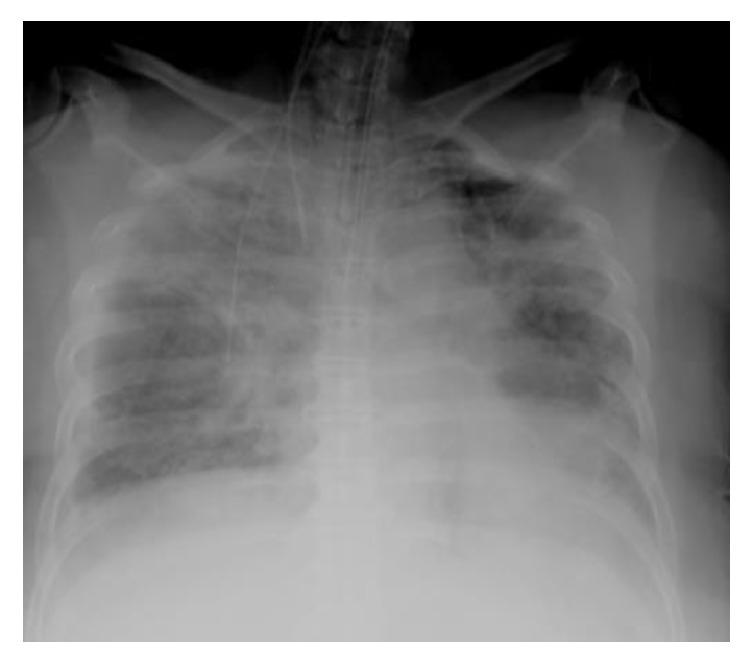
X-ray before transplant.

A chest X-ray performed after line placement (Figure 1) demonstrated satisfactory right internal jugular placement with the tip of the catheter positioned in the superior vena cava and without any evidence of pneumothorax. The liver transplant was performed uneventfully. Cold ischemic time was 7 hours and 3 minutes, warm ischemic time was 32 minutes, and portosystemic bypass time was 1 hours and 9 minutes.

**Figure 2:**
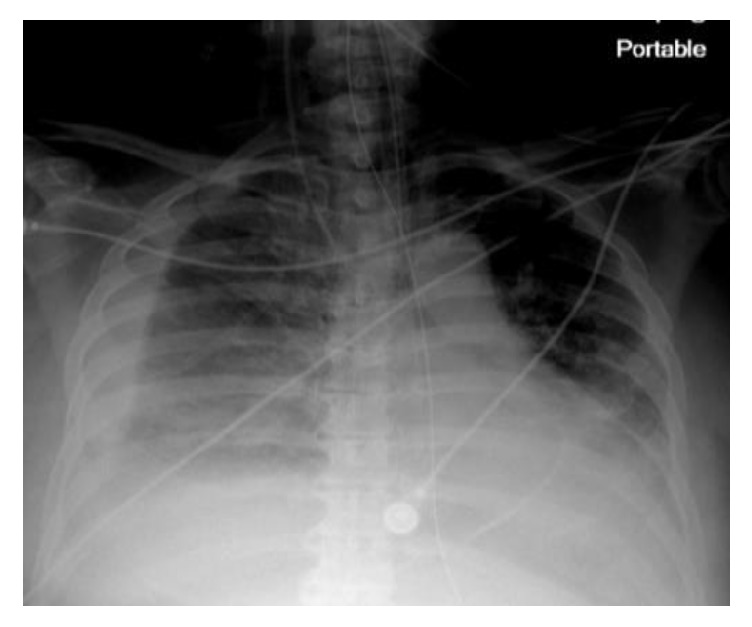
Post-op X-ray showing a foreign catheter tip in the heart.

**Figure 3:**
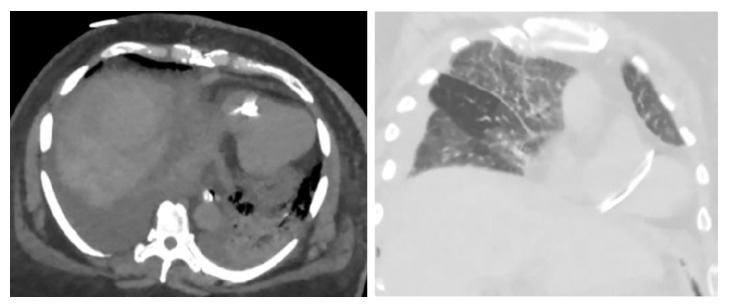
CT chest showing the fractured catheter in the right ventricle.

**Figure 4:**
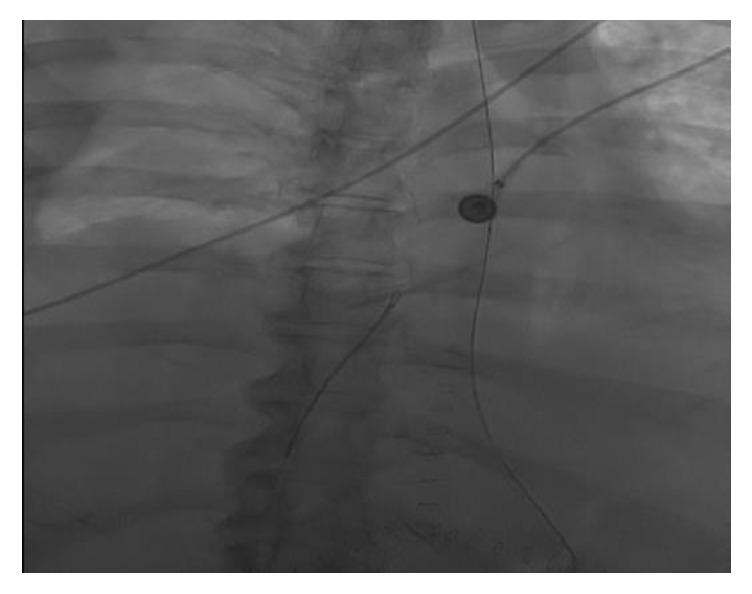
Catheter retrieval via snare device under fluoroscopy.

A routine postoperative chest X-ray (Figure 2) displayed a linear density, representing a catheter fragment, at the level of the right ventricle. The patient remained intubated and hemodynamically stable, with no evidence of jugular venous distension. Cardiac monitoring demonstrated sinus tachycardia and cardiac enzymes were negative. A Chest CT (Figure 3) was performed and demonstrated a fragment of distal portion of the catheter projecting at the apical wall of the right ventricle. The internal jugular central line catheter placed by anesthesiologist was removed and careful examination found the catheter to be complete and intact. The intracardiac catheter remnant was successfully retrieved by interventional radiology team under fluoroscopy, using 5 mm trilobed snare device through the right femoral vein (Figure 4). Examination of the foreign body revealed an 8 cm portion of a triple lumen catheter with a clean 45-degree cut at its distal end.

Further investigation determined that the foreign central line catheter placed in the donor was inadvertently transected, with embolization to the donor hepatic vein through donor IVC, by the cardiac transplant team during procurement of the donor's heart and lungs. The liver transplant with subsequent reperfusion resulted in the dislodgement and embolization of the remnant into the right ventricle of the recipient.

The patient continued to recover well and was extubated on postoperative day 1. Following continued recovery, on postoperative day 5, patient was discharged to rehabilitation center.

## 3. Discussion

Intravascular embolization of catheter fragments is a rare entity that was first described as a complication of central venous catheter placement in 1954 by Aitken and Minton as they described a case of catheter fracture and embolization secondary to a pinching effect between the clavicle and the first rib [4]. Due to the scarcity of this rare complication, the literature is mostly based on small series and case reports. Lin et al. reported rates of port rupture of 2.17% [5]. Surov et al. reported catheter fracture and embolization in a total of 215 patients in an extensive literature search from 1985 to 2007; of these 215 cases, 143 (66.5%) were implanted venous devices or port catheters and the remaining 72 (33.5%) were percutaneous venous catheters (PVC) with an extracorporeal portion [6].

Several mechanisms can trigger catheter fracture and embolism. The most common cause of fracture appears to be from the “Pinch-off syndrome” in which the catheter in implantable venous device or port access is subjected to recurrent compression and narrowing as the catheter passes over the first rib and beneath the clavicle leading to impingement against a vein wall and chronic intermittent compression of the device [7]. Other potential causes of catheter fracture include technically difficult implantation or explantation, forced insertion of catheter, and difficult removal of catheters that are entangled in fibrosis formation around the catheter. Difficulties during insertion or extraction should prompt for further examination of possible injury. In addition, catheter material fatigue from prolonged use contributes to in situ fracture, fragmentation, and distal embolization [8].

Traumatic catheter fracture has been described in several case reports. Kumar et al. reported a case of an accidental port catheter fracture attributed to a closed trauma of the shoulder [9], while Shailendra Deep et al. described a rare complication of transection of internal jugular line during hair cutting resulting in lodging of the line within the internal jugular vein [10].

The clinical presentation of catheter embolization varies considerably ranging from an asymptomatic incidental finding on a chest X-ray to serious life-threatening complications such as myocardial perforation, arrhythmias or septic emboli [11, 12]. As transection occurs, catheter fragment often migrates distally and finally lodges in the vena cava, right atrium, right ventricle, pulmonary artery, or its branches. The length, weight, and the material stiffness often determine the final lodgment site and its consequence symptoms [13].

The managements of dislodged or fractured central venous port catheter include percutaneous transcatheter retrieval or open thoracotomy retrieval. Among these, percutaneous retrieval of the dislodged catheter offers an easy and efficient approach without the increased morbidity of a thoracotomy; concurrent use of pigtail and loop snare catheters is a feasible way for percutaneous retrieval of a dislodged central venous catheter as this has even become a standard treatment of choice for catheter retrieval [14].

## 4. Conclusion

Literature searches revealed that most of the catheter embolization occurs in either the oncological or pediatric patient population, directly related to the prolonged use of central venous access that will lead to its impingement, material degeneration, and eventual fracture.

Inadvertent traumatic transection of the catheter is rare entity that is only described in few case reports. We did not find any similar case emphasizing transection and migration of catheter fragments to a different patient via a transplanted organ.

In order to prevent similar complication in the future we strongly suggest a careful examination of all venous access catheters before and after organ procurement in order to prevent similar complication in the future. We also suggest repeated flushing of all venous vasculatures with Wisconsin solution after organ procurement to flush out any remnant fragments and thereby preventing similar embolization of remnant catheters.
